# Evaluation of different DNA extraction methods and loop-mediated isothermal amplification primers for the detection of *Mycobacterium ulcerans* in clinical specimens

**DOI:** 10.1186/s12879-021-06308-z

**Published:** 2021-06-23

**Authors:** Anthony Ablordey, Evans Ahotor, Charles A. Narh, Sandra A. King, Isra Cruz, Joseph M. Ndung’u, Dziedzom K. de Souza

**Affiliations:** 1 Noguchi Memorial Institute for Medical Research, College of Health Sciences, University of Ghana, Legon, Accra, Ghana; 2 Foundation for Innovative New Diagnostics, Geneva, Switzerland; 3 National School of Public Health, Instituto de Salud Carlos III, Madrid, Spain

**Keywords:** Loop mediated isothermal amplification, DNA extraction, Sensitivity, Specificity

## Abstract

**Background:**

Early diagnosis and treatment of Buruli ulcer is critical in order to avoid the debilitating effects of the disease. In this regard, the development of new diagnostic and point of care tools is encouraged. The loop-mediated isothermal amplification for the detection of *Mycobacterium ulcerans* represents one of the new tools with a good potential of being developed into a point of care test. There is however the need to standardize the assays, reduce sample preparation times, improve the detection/visualization system and optimize them for high-throughput screening, adaptable to low resourced laboratories.

**Methods:**

In this study, we assessed two DNA extraction protocols (modified Boom and EasyNAT methods), three previously published LAMP primer sets (BURULI, MU 2404 and BU-LAMP), and compared the sensitivity and specificity of LAMP assays on three DNA amplification platforms.

**Results:**

Our results show that Buruli ulcer diagnosis using primers targeting *IS2404* for the LAMP method is sensitive (73.75–91.49%), depending on the DNA extraction method used. Even though the modified Boom DNA extraction method provided the best results, its instrumentation requirement prevent it from being field applicable. The EasyNAT method on the other hand is simpler and may represent the best method for DNA extraction in less resourced settings.

**Conclusions:**

For further work on the development and use of LAMP tests for Buruli diagnosis, it is recommended that the BURULI sets of primers be used, as these yielded the best results in terms of sensitivity (87.50–91.49%) and specificity (89.23–100%), depending on the DNA extraction methods used.

## Background

Buruli ulcer (BU) caused by *Mycobacterium ulcerans* is a necrotizing skin disease endemic in several rural communities in sub-Saharan Africa, particularly in Cote d’Ivoire, Ghana and Benin. There are also other foci of BU in the Americas, as well as in Asia and Oceania, with a very sharp recent increase in the number of cases in Australia in recent years. BU is the third most prevalent mycobacterial disease after tuberculosis [1–3]. Due to poor understanding of its epidemiology and transmission, there are no effective primary preventive measures for control of the disease. Control efforts focus on early case detection and prompt treatment to stop progression to the ulcerative stages, and thus avoid the associated disabilities [4].

Absence of effective diagnostic services accounts for the poor surveillance of the disease [5, 6]. In rural areas where majority of cases are detected, samples are often sent to reference laboratories for confirmatory testing, which takes 1–2 weeks. District hospitals rely on clinical diagnosis, but this is often inaccurate and complicated by other disease conditions with similar presentations, leading to misdiagnosis. Microscopic examination detects 29–78% of clinically suspected BU cases and is currently the only rapid and affordable test available for BU diagnosis in many endemic areas [6]. The detection rate of culture is between 34 and 79% and takes an average of 9–12 weeks to yield positive results but this too is only available in reference labs [6]. Culture therefore cannot be used for rapid laboratory confirmation. Histopathological examinations reportedly detect 30% additional cases than other confirmatory tests, however these techniques are restricted to external reference laboratories and are unavailable in peripheral health centres, district or regional hospitals [6]. These challenges; including poor conditions for sample transportation, and long turnaround times, contribute to late treatment and management of the disease. The WHO’s Second International Conference on Buruli Ulcer Control and Research resolved to strengthen the capacity of national laboratories to confirm cases of the disease but warned that “efforts are still needed to develop simple diagnostic tools usable in the field as well as disability prevention methods” [7]. Therefore, new field applicable techniques for the diagnosis of BU, paralleling PCR in sensitivity and specificity, need to be developed [2, 6].

IS*2404* PCR has close to 96% sensitivity and has been recommended by WHO for laboratory confirmation of BU [8–13]. However, PCR is labour intensive, expensive and unsuited for use in rural African settings with poorly resourced labs [14]. Isothermal amplification of nucleic acids in recent times has been relied upon to circumvent the limitations of traditional PCR and provides a platform that can support the development of rapid Point of Care (POC) molecular diagnostic tests [15]. Loop-mediated isothermal amplification (LAMP) is a method that amplifies DNA with high specificity, sensitivity and rapidity at a constant temperature. The method utilizes the strand displacement ability of the *Bacillus stearothermophilus* DNA polymerase (*Bst* polymerase) to loop 4–6 primer pairs, including inner and outer primers, to the target loci of interest. Thus, it yields several stem-loop amplicons, which sustains the reaction cycling at 60–65 °C. The main advantage of LAMP over PCR is that it does not require thermal cyclers and gel imaging equipment, and thus, amplification can be performed within an hour or less in a laboratory with a heat block or water bath [8, 16]. LAMP reaction results can be visualised with the naked eye from colorimetric changes following addition of intercalating fluorescent dyes such as SYBR green or hydroxynapthol blue (HNB) to reaction tubes, pre- or post-reaction [17]. LAMP is also less inhibited by contaminants within the sample and the closed tube approach reduces problems of cross contamination. It has been widely developed for detection of various disease pathogens, including viral, bacterial and protozoan [18–22]. The superiority of LAMP, cost-effectiveness and ease of use by potential ‘unskilled’ personnel makes it an attractive molecular diagnostic tool to further develop for clinical use, particularly in resource-limited settings where PCR cannot be afforded.

Previous studies developed LAMP assays for the detection of *Mycobacterium ulcerans* in clinical samples [8, 16, 23]. Separately, two of the primers (Table 1) amplified fragments of the multi-copy insertion sequence, IS*2404,* of which 214 copies are present in the genome of the bacterium [8, 23], while the other amplified another insertion sequence, *IS2606*, with about 60 genomic copies present [16]. The results showed superior sensitivity of LAMP to PCR [8, 16, 23]. However, the sample sizes used in these investigations were small and different methods were used, which did not allow accurate comparisons of the data. Hence, there was a need to standardize the assays, reduce sample preparation times, improve the detection/visualization system, and optimize them for high-throughput screening, adaptable to low resourced laboratories. To reduce the turnaround times, we utilized and compared two rapid DNA extraction protocols for sample preparation. Using several clinical samples from endemic communities, we compared the sensitivity and specificity of LAMP assays on three DNA amplification platforms. Three recommended fluorescent dyes were also optimized and used to visualize test results. We present comparative genomic DNA extraction and primer-specific LAMP data that provide suitable alternatives with remarkable sensitivity for rapid diagnosis of BU.

## Methods

### Work flow

**Fig. 1:**
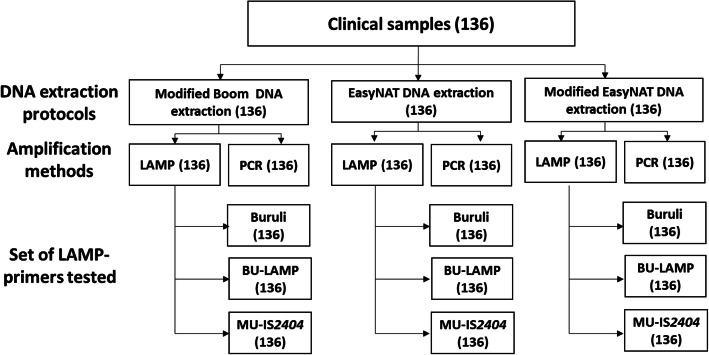
Flow diagram of study procedure showing assays performed and the total number of samples tested

The flow diagram of the study is shown in Fig. 1.

### Samples

The samples used in this study were archived as well as fresh patient’s samples. Fresh samples were collected from consenting patients attending Buruli ulcer Clinic at selected district hospitals and health centres in Ghana, using the Fine Needle Aspiration (FNA) and Swabs methods [24, 25]. The Noguchi Memorial Institute for Medical Research (NMIMR) is one of the two reference centres in Ghana where patients’ samples are sent for confirmation. As such, these samples were also included in the study. Swabs and FNAs were respectively put into sterile 15 ml falcon tubes and phosphate buffered saline, and transported in cool box to NMIMR.

### DNA extractions

Previous results indicated that crude DNA preparations yield low sensitivity compared with purified extracts in the LAMP assay [8]. Consequently, crude DNA extracts were not included in this work. In order to simplify DNA extraction for the LAMP test in field settings, two simple and rapid extraction protocols were used; a commercially available syringe silica membrane method (EasyNAT) for the extraction of *M. tuberculosis* DNA from sputum, and a modification of the syringe silica method (modified EasyNAT).

#### Modified Boom extraction

Portions (250 μl) of the sample suspension were lysed with 250 μl of lysis buffer (1.6 M guanidinum HCl, 60 mM Tris (pH 7.4), 1% Triton X-100, 60 mM EDTA, 10% Tween-20), 50 μl of Proteinase K (20 mg/ml) and 500 μl glass beads in a sterile 1.5 ml tube. The mixture was incubated horizontally in an orbital shaker (200 rpm) at 60 °C for 12 h. To capture the DNA, 40 μl of diatomaceous earth solution was added to the lysed sample and incubated at 37 °C for 1 h. The mixture was then centrifuged at 14000 rpm for 1 min and the supernatant discarded. Pelleted DNA bound to the diatomaceous earth was washed twice with 900 μl of ice cold 70% ethanol and 900 μl of absolute acetone, pulsed vortexed and centrifuged at 14000 rpm for 60s. The supernatant was discarded and DNA bound to diatomaceous earth was dried at 50 °C for 20 min using a heat block. DNA was re-suspended in 80 μl of nuclease free water, incubated at 68 °C for 20 min to release the DNA from the diatomaceous earth. The DNA extract was centrifuged at 14000 rpm for 2 min and about 60 μl of the resultant supernatant transferred to a sterile tube and kept at -20 °C. Negative and positive controls were included in each batch of DNA extraction. The negative control included all the reagents for DNA extraction, but excluding a clinical specimen, while the positive control included 250 μl of *M. ulcerans* culture in suspension.

#### Genomic DNA extraction using disposable silica syringe DNA extraction device (EasyNAT)

DNA was extracted from suspension of clinical samples following the instructions provided by the manufacturer (USTAR Biotechnologies Ltd., Hangzhou, China). Briefly, 500 μl of clinical specimen was pipetted into 1.5 mL tube, then 600 μl of lysis buffer and 50 μl Proteinase K were added. The mixture was incubated at 60 °C for 30 min and allowed to cool to room temperature. 1400 μl of absolute ethanol was added to the lysed clinical specimen, and the tube was inverted 3–4 times. The mixture was aspirated through the nozzle of an assembled silica syringe device (SSD), with the aid of the attached plunger. The lysed sample was slowly sucked back into the tube, leaving the DNA bound to the silica membrane at the base of the syringe. The DNA bound to the silica membrane was washed twice by sucking 60% of absolute ethanol through the nozzle of the syringe and slowly expelled. At this step, the DNA remained bound to the silica membrane. The 5 ml syringe barrel was detached from the SSD and replaced with a 1 ml syringe barrel (included in the kit). 100 μl of nuclease free water was sucked and allowed to soak the silica membrane for 2 min. The dissolved DNA was eluted into a 1.5 ml tube.

#### Genomic DNA extraction using a modified EasyNAT protocol

Suspension of clinical sample (150 μl) was lysed by adding 600 μl of lysis buffer (1.6 M guanidinum HCl, 60mMTris (pH 7.4), 1% Triton X-100, 60 mM EDTA, 10% Tween20) and 50 μl Proteinase K (20 mg/ml). The mixture was incubated at 60 °C for 30 min and allowed to cool to room temperature. Absolute ethanol (1400 μl) was added to the lysed sample, vortexed and sucked through the assembled silica syringe device (SSD) with the aid of the plunger. The lysed sample was then filtered back to the tube by pushing the plunger slowly. DNA bound to the silica membrane at the base of the SSD was washed twice with 2 ml of 60% alcohol using air pressure generated from pushing the plunger. The 5 ml syringe barrel was detached from the SSD and replaced with 1 ml syringe barrel for DNA elution. Nuclease free water (100 μl) was then sucked through the nozzle of the syringe. With the aid of the plunger, nuclease free water was sucked over the silica membrane 3–4 times and finally eluted in a 1.5 ml eppendorf tube.

### Nucleic acid amplifications

All DNA samples were tested with *IS2404* PCR reference method [13]. The primer sequences for the IS*2404* PCR is shown in Table 1. All published LAMP primer sets (Table 2) were tested on the different DNA extracts, in order to determine their sensitivity. LAMP assays were performed using the Loopamp DNA amplification kit (Eiken Chemical). For specificity, the primers were also tested on *M. nonchromogenicum, M. fluoranthenivorans, M. fortuitum, M. porcinum, M. mucogenicum, M. ulcerans,* and *M. tuberculosis*.

**Table 1:** IS*2404* nested PCR primers

Primers	Sequence (5′-3′)	Expected Amplicon size (bp)
pGp1	AGGGCAGCGCGGTGATACGG	518
pGp2	CAGTGGATTGGTGCCGATCGAG	
pGp3	GGCGCAGATCAACTTCGCGGT	212
pGp4	CTGCGTGGTGCTTTACGCGC

**Table 2:** LAMP primers from the three different studies

**Ablordey et al, 2012** [8]	
Buruli-FIP:GTGCGCCGTGTCCGGTATGGATACGCGATGTCACCTTC	
Buruli- BIP: AGGTCCTAGCAACGCTACGCAAATCCGGCAGGCTTCGG	
Buruli-LF: GCCTTTGACGGTCTTCGTC	
Buruli- LB: CACCGCGATCAATCTGCAC	
Buruli- F3: CGAGAACAGCCTGCACTG	
Buruli- B3: CGGTTGGCGGTCAAAGC	
**Njiru et al, 2012** [23]	
MU2404-FIP: CGTCGCGTATCCAGTGCAGGTCATGACCTGGATGCGTCA	
MU2404-BIP: GCGCACAGGTCCTAGCAACGGCTTCGGCGATGTTGTCG	
MU2404-F3:GCCTGCCGTTCGAGCA	
MU2404-B3:GTCAAAGCGGTGATCCGG	
MU2404-FLP:TTCTCGATTCCGCAGTG	
MU2404-BLP:GCAACACCGCGATCAATCTG	
**de Souza et al, 2012** [16]	
BU-LAMP FIP: GCATCTCCGGCCACCCCAACGCCCAACGACCGCTA	
BU-LAMP BIP:GTGGTGGGCCCCTGGGAAACCGCTGTCGAACTGTGC	
BU-LAMP F3: ACGGATCGTCGAGGATGG	
BU-LAMP B3:GCGCCAGGTCCCTTGA	
BU-LAMP FLP:GAGCCTGCTGGGCGGTC	
BU-LAMP BLP: CAGATCCCACCCTGGTG	

### Preparation of suspensions containing different copy numbers of IS*2404* for determination of the detection limit of the LAMP tests

A single colony of *M. ulcerans* growing on Lowenstein-Jensen media slant was harvested in nuclease free water. DNA was extracted from this culture using the QIAGEN DNeasy Blood & Tissue Kits (QIAGEN, California). The concentration of the final extract was 0.2 ng/μl (stock concentration in a final volume of 20 μl), which was equivalent to 300,000 copies of IS*2404*. The latter was serially diluted in a volume of 20 μl to contain 30,000, 3000, 300, 30 and 3 copies of IS*2404*. LAMP assays were performed on these dilutions to measure the detection limit of the three primer sets.

### Optimization of visualization system for *M. ulcerans*

The detection of LAMP amplicons can utilize several intercalating fluorescent dyes [17], which suggests that an optimum dye concentration will increase the detection limit. In this study, we also compared two dyes; Calcein and HNB using protocols described elsewhere [17, 26]. This was done in closed tubes to prevent possible cross-contamination. Calcein turns from yellow (negative test) to fluorescent green (positive test) and hydroxynapthol blue turns from violet (negative test) to sky blue (positive test).

### Data analysis

For data analysis, 2 × 2 contingency tables were generated for the evaluation of diagnostic accuracy. The data was analysed for diagnostic test evaluation using MedCalc Software (Version 18.6). The disease prevalence, sensitivity, specificity, positive and negative predictive values as well as accuracy, were expressed as percentages, with their corresponding 95% confidence intervals.

## Results

### Comparison of DNA extraction protocols

Results of IS*2404* PCR performed on the three different DNA extracts is shown in Table 3 below. A total of 136 extracts were analysed. The modified Boom DNA extraction method gave the highest PCR positivity (69.12%), followed by the EasyNAT DNA extraction method (58.82%).

**Table 3:** PCR analysis of 136 clinical samples. Values in brackets represent the 95% CI

Sample	PCR on Modified Boom DNA Extract		PCR on Modified EasyNAT DNA extract		PCR on EasyNAT DNA extract	
	+ve	-ve	+ve	-ve	+ve	-ve
136	94	42	71	65	80	56
Positivity %	69.12 (60.63–76.75)		52.21 (43.48–60.84)		58.82 (50.07–67.19)	

### Sensitivity and specificity of LAMP assays in combination with different DNA extracts

The results of the various tests were compared for their sensitivity, specificity and predictive values and accuracy (Table 4). For this, the *IS2404* PCR was used as the reference standard. For a particular LAMP assay and with a particular DNA extract, more positives were detected with the BURULI primer set designed by Ablordey [8], followed by the MU 2404 primers of Njiru [23] and then MU LAMP primers of de Souza [16] (Table 4). Specificity however did not follow a similar pattern. Across corresponding tests, the LAMP test on Boom extracts yielded the most number of positives followed by EasyNAT and then the modified EasyNAT extracts. However positivity of the LAMP test for modified and unmodified extracts where the same. For the modified Boom, EasyNAT and modified DNA extracts, the BURULI primers yielded sensitivities of 91.49, 87.50 and 90.14%, and specificities of 97.26, 100.00 and 89.23% respectively. The MU 2404 primers yielded sensitivities of 86.12, 73.75, 83.10% and specificities of 88.10, 98.21 and 93.85% respectively. The BU-LAMP primers on the other hand yielded the lowest sensitivities of 57.45, 47.50, 35.21% and highest specificities of 100.00, 98.21 and 95.38% respectively. Thus, comparing the trade off between sensitivity and specificity, coupled with the predictive values and the accuracies, the BURULI primers gave the best results.

**Table 4:** Comparison of sensitivity and specificity of three LAMP tests based on calcein visualization with different DNA extracts. Values in brackets represent the 95% CI

	**LAMP WITH MODIFIED BOOM DNA EXTRACTS**								
	**BURULI Primers**			**MU 2404 Primers**			**BU-LAMP Primers**		
	**(+)**	**(−)**	**Total**	**(+)**	**(−)**	**Total**	**(+)**	**(−)**	**Total**
**IS*2404*** **PCR (+)**	86	8	94	81	13	94	54	40	94
**IS*2404*** **PCR (−)**	1	41	42	5	37	42	0	42	42
**Total**	87	49	136	86	50	136	54	82	136
**Disease prevalence**	69.12% (60.63 to 76.75%)			69.12% (60.63 to 76.75%)			69.12% (60.63 to 76.75%)		
**Sensitivity**	91.49% (83.92 to 96.25%)			86.17% (77.51 to 92.43%)			57.45% (46.82 to 67.59%)		
**Specificity**	97.62% (87.43 to 99.94%)			88.10% (74.37 to 96.02%)			100.00% (91.59 to 100.00%)		
**Positive Predictive Value**	98.85% (92.53 to 99.83%)			94.19% (87.64 to 97.37%)			100%		
**Negative Predictive Value**	83.67% (72.50 to 90.88%)			74.00% (62.93 to 82.67%)			51.22% (45.36 to 57.04%)		
**Accuracy**	93.38% (87.81 to 96.93%)			86.76% (79.89 to 91.96%)			70.59% (62.17 to 78.09%)		
	**LAMP WITH EasyNAT DNA EXTRACTS**								
	**BURULI Primers**			**MU 2404 Primers**			**BU-LAMP Primers**		
	**(+)**	**(−)**	**Total**	**(+)**	**(−)**	**Total**	**(+)**	**(−)**	**Total**
**IS*2404*** **PCR (+)**	70	10	80	59	21	80	38	42	80
**IS*2404*** **PCR (−)**	0	56	56	1	55	56	1	55	56
**Total**	70	66	136	60	76	136	39	97	136
**Disease prevalence**	58.82% (50.07 to 67.19%)			58.82% (50.07 to 67.19%)			58.82% (50.07 to 67.19%)		
**Sensitivity**	87.50% (78.21 to 93.84%)			73.75% (62.71 to 82.96%)			47.50% (36.21 to 58.98%)		
**Specificity**	100.00% (93.62 to 100.00%)			98.21% (90.45 to 99.95%)			98.21% (90.45 to 99.95%)		
**Positive Predictive Value**	100%			98.33% (89.39 to 99.76%)			97.44% (84.31 to 99.63%)		
**Negative Predictive Value**	84.85% (75.82 to 90.91%)			72.37% (64.42 to 79.11%)			56.70% (51.46 to 61.80%)		
**Accuracy**	92.65% (86.89 to 96.42%)			83.82% (76.54 to 89.58%)			68.38% (59.86 to 76.08%)		
	**LAMP WITH MODIFIED EasyNAT DNA EXTRACTS**								
	**BURULI Primers**			**MU 2404 Primers**			**BU-LAMP Primers**		
	**(+)**	**(−)**	**Total**	**(+)**	**(−)**	**Total**	**(+)**	**(−)**	**Total**
**IS*2404*** **PCR (+)**	64	7	71	59	12	71	25	46	71
**IS*2404*** **PCR (−)**	7	58	65	4	61	65	3	62	65
**Total**	71	65	136	63	73	136	28	108	136
**Disease prevalence**	52.21% (43.48 to 60.84%)			52.21% (43.48 to 60.84%)			52.21% (43.48 to 60.84%)		
**Sensitivity**	90.14% (80.74 to 95.94%)			83.10% (72.34 to 90.95%)			35.21% (24.24 to 47.46%)		
**Specificity**	89.23% (79.06 to 95.56%)			93.85% (84.99 to 98.30%)			95.38% (87.10 to 99.04%)		
**Positive Predictive Value**	90.14% (81.89 to 94.87%)			93.65% (85.02 to 97.46%)			89.29% (72.53 to 96.34%)		
**Negative Predictive Value**	89.23% (80.32 to 94.39%)			83.56% (75.15 to 89.52%)			57.41% (52.97 to 61.73%)		
**Accuracy**	89.71% (83.33 to 94.26%)			88.24% (81.60 to 93.12%)			63.97% (55.30 to 72.02%)		

Across corresponding tests, sensitivity was highest for the LAMP test on Boom extracts (57.45–91.49%), followed by the modified EasyNAT (35.21–90.14%) and then unmodified EasyNAT extracts (47.50–87.50%). Specificity did not follow a specific pattern, but the values were high (88.1–100%). The results also showed the EasyNAT method to have a high specificity (98.2–100%) i.e. the probability of screening negative if the disease is truly absent. On the other hand, the modified EasyNAT method had the lowest sensitivities (35.21–90.14%) i.e. the probability of screening positive if the disease is truly present. The predictive values of the various tests were also estimated. The positive predictive value, which estimates the probability that an individual actually has the disease given a positive test, varied according to the methods.

The detection limit of the LAMP test was 30 copies of IS*2404*. However half and a quarter of the tests were positive for BURULI and MU 2404 primers respectively (Table 5). Concerning the specificity of the tests, the results revealed that, of the eight *Mycobacterium species* tested with the three sets of primers, only *M. ulcerans* isolates tested positive in the LAMP test.

**Table 5:** Detection limit of the LAMP tests with DNA extracted using QIAGEN, and visualized based on calcein detection system

IS***2404*** copy numbers	Number positive per test		
	BURULI Primers	MU 2404 Primers	BU-LAMP Primers
300,000	1/1	1/1	1/1
30,000	1/1	1/1	¼
3000	1/1	1/1	0/5
300	2/3	2/3	0/5
30	2/4	1/4	0/5
3	0/4	0/4	0/5

### Comparison of fluorescence detection dyes

The optimization of a fluorescent dye system was undertaken using the BURULI and MU 2404 primers. For a given test (BURULI or MU 2404), no significant difference was found between tests performed using calcein or HNB. (Table 6).

**Table 6:** Comparison of LAMP reactions using two different dyes, on DNA extracted using QIAGEN. Values in brackets represent the 95% CI

	BURULI Primers						MU 2404 Primers					
	Calcein			HNB			Calcein			HNB		
	+	–	Total	+	–	Total	+	–	Total	+	–	Total
*IS2404* (+)	54	8	62	55	7	62	54	8	62	57	5	62
*IS2404* (−)	1	42	43	2	41	43	4	39	43	4	39	43
Total	55	50	105	57	48	105	58	47	105	61	44	105
Positivity (%)	51.4			52.4			51.4			54.3		
Sensitivity (%)	87.10 (76.15 to 94.26)			88.71 (78.11 to 95.34)			87.10 (76.15 to 94.26)			91.94 (82.17 to 97.33)		
Specificity (%)	97.67 (87.7 to 99.94)			95.35 (84.19 to 99.43)			90.70 (77.86 to 97.41)			90.70 (77.86 to 97.41)		
PPV (%)	98.18 (88.59 to 99.73)			96.49 (87.63 to 99.07)			93.10 (84.08 to 97.18)			93.44 (84.82 to 97.32)		
NPV (%)	84.00 (73.30 to 90.94)			85.42 (74.40 to 92.19)			82.98 (71.71 to 90.36)			88.64 (77.00 to 94.78)		
Accuracy (%)	91.43 (84.35 to 96.01)			91.43 (84.35% to 96.01)			88.57 (80.89 to 93.95)			91.43 (84.35 to 96.01)		

## Discussions

This study revealed that the efficiency of the LAMP reaction depends on the quality of extracted DNA and the efficiency of the primers used. We compared the sensitivities and specificities of the various tests, as well as their predictive values, using *IS2404* PCR as the gold standard. In deciding upon the criteria for acceptable levels of specificity and sensitivity, it is important to consider the consequences of leaving cases undetected (false negatives) against the false classification of healthy individuals as infected (false positives) [27]. In the context of BU diagnosis, in the absence of other options, the sensitivity of a diagnostic method should be increased at the expense of specificity, since the penalty associated with missing a case is high. Our results indicated that the modified Boom extraction method had the highest sensitivities. Thus, it is a good method for use in reference laboratories. However, the appropriateness of this method for confirmation of Buruli ulcer cases in resource limited settings should be cautiously explored, due to the requirements associated with it. It is not very practical for use in resource limited settings as it requires complex, lengthy procedures and sophisticated equipment. Thus, in terms of programmatic feasibility, testing requirements, time and cost, the modified EasyNAT method appears to be the best for field monitoring and evaluation programs, with sensitivities of 90.14 and 83.10% for the BURULI and MU 2404 primers respectively. These results also showed the BURULI to be best suited for analysis, since these gave the highest sensitivities, best predictive values and accuracies.

The aforementioned primers are both based on *IS2404*, while BU-LAMP primers are based on *IS2606*. PCR assays based on *IS2404* and *IS2606* revealed that *IS2606* PCR was 10 times less sensitive, compared to *IS2404* PCR [13]. The copy numbers for the IS*2404* and IS*2606* target sequences in the genome of *M. ulcerans* strain Agy99 are 213 and 91 respectively [28], representing a little over twice as many copies of *IS2404* compared to *IS2606*. These differences in copy numbers may explain why the IS*2404* based primers resulted in a higher sensitivity.

## Conclusions

The need for confirmation of Buruli ulcer cases requires tools that are sensitive. The results of this work would permit the development of improved tools for the diagnosis for Buruli ulcer. These assays, if adapted for field settings and poorly resourced laboratories, have the robustness and good turnaround times, to be used by ‘semi-skilled’ lab technicians with little supervision. For the development and use of future tests for Buruli diagnosis using the LAMP method, it is recommended that the BURULI primers [8] be used.

## Data Availability

The datasets used and/or analysed during the current study are available from the corresponding author on reasonable request.

## Abbreviations

DNA: Deoxyribonucleic acid
LAMP: Loop mediated isothermal amplification
MU: *Mycobacterium ulcerans*
BU: Buruli ulcer
PCR: Polymerase chain reaction
SSD: Silica syringe device
HNB: Hydroxynapthol blue
EDTA: Ethylenediaminetetraacetic acid
NMIMR: Noguchi memorial institute for medical research
